# Remote working in public involvement: findings from a mixed methods study

**DOI:** 10.1186/s40900-022-00396-0

**Published:** 2022-11-04

**Authors:** Elisa Jones, Lucy Frith, Mark Gabbay, Naheed Tahir, Muhammad Hossain, Mark Goodall, Katie Bristow, Shaima Hassan

**Affiliations:** 1 Department of Primary Care and Mental Health, University of Liverpool, Liverpool, UK; 2 Centre for Social Ethics and Policy, University of Manchester, Manchester, UK; 3 National Institute for Health and Care Research ARC North West Coast, Liverpool, UK; 4 Health and Social Care, University of Wales Trinity Saint David, Lampeter, UK

**Keywords:** Public patient involvement and engagement, Health inequalities, Covid-19 pandemic, Remote working, Digital literacy, Online video conferencing

## Abstract

**Background:**

This paper considers remote working in patient public involvement and engagement (PPIE) in health and social care research. With the advent of the Covid-19 pandemic and associated lock-down measures in the UK (from March 2020), PPIE activities switched to using remote methods (e.g., online meetings), to undertake involvement. Our study sought to understand the barriers to and facilitators for remote working in PPIE by exploring public contributors’ and PPIE professionals’ (people employed by organisations to facilitate and organise PPIE), experiences of working remotely, using online and digital technologies. A particular focus of our project was to consider how the ‘digital divide’ might negatively impact on diversity and inclusion in PPIE in health and social care research.

**Methods:**

We used a mixed method approach: online surveys with public contributors involved in health and social care research, online surveys with public involvement professionals, and qualitative interviews with public contributors. We co-produced the study with public contributors from its inception, design, subsequent data analysis and writing outputs, to embed public involvement throughout the study.

**Results:**

We had 244 respondents to the public contributor survey and 65 for the public involvement professionals (PIPs) survey and conducted 22 qualitative interviews. Our results suggest public contributors adapted well to working remotely and they were very positive about the experience. For many, their PPIE activities increased in amount and variety, and they had learnt new skills. There were both benefits and drawbacks to working remotely. Due to ongoing Covid restrictions during the research project, we were unable to include people who did not have access to digital tools and our findings have to be interpreted in this light.

**Conclusion:**

Participants generally favoured a mixture of face-to-face and remote working. We suggest the following good practice recommendations for remote working in PPIE: the importance of a good moderator and/or chair to ensure everyone can participate fully; account for individual needs of public contributors when planning meetings; provide a small expenses payment alongside public contributor fees to cover phone/electricity or WiFi charges; and continue the individual support that was often offered to public contributors during the pandemic.

## Plain English summary

This paper looks at remote working in patient public involvement and engagement (PPIE) in health and social care research. When the Covid-19 pandemic began and the UK went into lock-down in March 2020, PPIE activities began to use remote working methods, such as Zoom or Teams online meetings. We co-developed a study to understand the experiences of both public contributors and PPIE professionals, those who are employed to organise PPIE, of working remotely. We were particularly interested in how remote working might affect diversity and inclusion in PPIE in health and social care research. We ran online surveys for public contributors and public involvement professionals and conducted semi-structured interviews with public contributors. We co-produced the study with public contributors to embed public involvement throughout the study. We had 244 respondents to the public contributor survey, 65 for the public involvement professionals survey and conducted 22 qualitative interviews. Due to ongoing Covid restrictions during the research project we could not include people who did not have access to digital tools, and this is a limitation of our project. We found that public contributors generally liked working remotely and, for many, their PPIE activities increased. There were both benefits and drawbacks to working remotely. From our findings, we have made a number of suggestions for how to run remote meetings in PPIE and what to prioritise based on the areas public contributors thought were important (such as one-to-one support).

## Introduction

This paper considers remote working in patient public involvement and engagement (PPIE) in health and social care research. With the advent of the Covid-19 pandemic and associated lock-down measures that started in March 2020 in the UK and continued into 2021, face-to-face meetings and events were no longer possible and PPIE switched to using remote working methods. The study on which this paper is based sought to understand the barriers *to* and facilitators *for* remote working in PPIE, by asking public contributors and PPIE professionals, those who are employed to facilitate and organise PPIE by organisations, about their experiences of and opinions about working remotely, using online and digital technologies. A particular focus of the project was to consider how the move to remote working in PPIE could affect the diversity of public contributors and how the ‘digital divide’ might negatively impact on diversity and inclusion in PPIE in health and social care research. This paper presents the results from this project and concludes with suggestions for developing good practice in remote working in PPIE.

## Background

PPIE has become a wide-spread phenomenon in health and social care research. The National Institute of Health and Care Research (NIHR) state: ‘Public involvement is at the centre of NIHR health and social care research, and the public have a right to have a say in what and how publicly funded research is undertaken.’ [19]. With the advent of the Covid pandemic, the NIHR reiterated its commitment to PPIE [19]. The terms ‘patient and public involvement and engagement’ (PPIE) or public patient involvement (PPI) are commonly used to capture a broad range of activities that aim to develop effective links between researchers and the general public. We will use a broad definition of PPIE for the purposes of this paper as, ‘research being carried out ‘with’ or ‘by’ contributors of the public rather than ‘to’, ‘about’ or ‘for’ them.’ [21]. PPIE includes notions of active contribution, and ‘good’ PPIE is more about co-production than just involvement (Hickey et al. [12]). ‘Co-producing a research project is an approach in which researchers, practitioners and the public work together, sharing power and responsibility from the start to the end of the project, including the generation of knowledge.’(Hickey et al. [12]) We will use the term ‘remote working’ to cover all types of public involvement that takes place remotely, whether it encompasses paid employment or not, and uses tools and platforms for non-face-to-face communication, such as telephones (land lines, mobiles, smart phones), online conferencing/meetings, social media, and apps.

We were interested in exploring how remote work might create particular challenges for ensuring access and engagement for public contributors. There is a digital divide that maps onto existing socio-economic and health inequalities [6], and it has been noted that PPIE conducted remotely has the potential to further disenfranchise already disadvantaged and marginalised groups [1]. Remote working is now becoming a mainstay of how meetings are conducted. The potential disenfranchisement due to the digital divide is, therefore, an ongoing issue and is added to concerns that PPIE was insufficiently diverse prior to the pandemic [23, 24]. A recent NIHR survey of public contributors found a lack of diversity in the public contributor community in terms of age and socio-economic status (NIHR), [20] and addressing this is a National Institute of Health Research priority (NIHR) [22], Therefore, considering how remote working in PPIE might affect diversity and inclusion in PPIE was a key aim of the study.

Within the context of wider initiatives that are seeking to address the increasing health inequalities caused by the pandemic, it is important to consider how we can organise and conduct remote working in PPIE [4]. With the easing of lockdown, remote working has given way to dual working, both remote and face-to-face; hence, some form of remote working is likely to continue. For example, ‘hybrid forms, with face-to-face and remote options are becoming more commonplace. Therefore, developing good remote working practices in PPIE is becoming increasingly important for all health and social care research. If done well, remote working, alongside face-to-face meetings, can make PPIE opportunities available to wider publics, i.e., those who cannot attend meetings physically due to complex social situations, disability and other potentially marginalised groups (such as refugees, young people) who may not be confident in unfamiliar environments. Thus, these new ways of involvement through remote working can be a space for change (Cornwall [5]). Remote working could also stimulate new ways of doing PPIE that are less focussed on traditional meeting formats and rely less on people having good literacy levels (Estacio [8]) and the increasing use of social media amongst healthcare communities [26].

There is limited research published on remote working in PPIE, which is unsurprising given the immediate requirement to work remotely prompted by the pandemic and hence the short timescale over which it became the dominant way of working. There were some instances of remote working in PPIE and prior to the pandemic, Brighton et al. [3] developed an online forum for PPI. Lampa et al. [16] reported their observations of digital PPIE meetings that had switched to remote online platforms due to the pandemic. They concluded that remote working was possible in PPIE but required commitment from researchers to work with contributors to solve practical issues. Adeyemi et al. [1] considered the challenges and adaptations which were needed for working remotely with marginalised groups during the pandemic.

Taking account ot these early findings from the advent of lockdown as PPIE moved online there was a growth in ‘how to’ guidance for conducting remote working in general and PPIE specifically. Organisations such as NIHR Research Design Service, NIHR School for Primary Care Research, and many public involvement teams produced introductions to Microsoft Teams, Zoom and other software to enable remote working and suggestions for how to manage and navigate PPIE activities in this new environment. Our project aimed to contribute both to the emerging literature on remote working specifically in PPIE and to provide some good practice recommendations for doing PPIE in this way.

## Methods

We used a mixed method approach, as having the breadth of responses from surveys and the ability to probe understanding in the qualitative interviews was an appropriate methodology for answering our research questions. The study began with quantitative surveys with public contributors involved in health and social care research and people who work professionally in public involvement, those who are employed to facilitate and organise PPIE by organisations. We then undertook purposively sampled qualitative interviews with public contributors to get a deeper insight into their experiences of remote working, until thematic saturation was achieved, that is until no new themes were emerging from the interviews. Finally, we used these findings to undertake a discrete choice experiment (reported elsewhere).

### Public involvement

We embedded public involvement and co-production in all stages of the project, from design through to delivery, analysis and writing up the findings. We co-developed the study with input from the ARC Public Advisor Forum, a group of 25–30 public contributors who meet regularly and advise, participate, and co-produce all the work of the ARC NWC. We had a public contributor as a funded co-applicant (NT) on the subsequent UKRI ESRC grant. We did not carry out any formal assessment of our PPIE activities, but held a number of workshops towards the end of the project to develop the short guidelines [2] and feedback on our involvement strategies.

### Survey design

We designed the surveys using Jisc Online Survey software. For the public contributor survey, we developed the questionnaire with our public contributor co-applicant (NT) and a group of ARC NWC public advisors. We also drew on our own experiences of conducting remote work in PPIE during the pandemic and the emerging literature in this area. Once the survey was developed, we piloted it with two public contributors, to check for sense, consistency and readability, refined the survey and then piloted it with the wider ARC NWC Public Advisors Forum, before rolling out nationally.

The survey comprised tick box questions, Likert scale questions, where participants could specify whether they agreed or disagreed with statements, and open-ended questions where participants could enter free text responses. The survey asked general questions about role and PPIE experience, digital literacy and different aspects of remote working. We collected demographic information to enable us to draw conclusions from the data on how age, ethnicity, living arrangements and socio-economic status impact on participants use of remote communication tools. The survey ran from September 2020 to February 2021.

We co-developed the survey for PPIE professionals, with input from our public contributors and PPIE professionals from the ARC NWC and the NIHR Research Design Service. We piloted the survey with members of the ARC team and public contributor (NT) to check for clarity, consistency and readability.

Like the PPIE contributor survey, the professional version was made up of: tick box, Likert scale and open-ended questions. We asked what support and training they offered their public contributors and any suggestions they had for improving remote working in PPIE. The survey ran from January to March 2021.

### Qualitative interviews

After the survey conducted with public contributors had closed, we purposively sampled informants aiming to interview some people from traditionally unrepresented groups in PPIE (such as people from minoritized backgrounds) and conducted 22 semi-structured qualitative interviews with public contributors from across the UK. The topic guide was co-developed with the research team and public contributor (NT) from a preliminary analysis of the survey results and was co-designed to probe and explore the issues raised by the survey. The interviews were conducted via Zoom; face-to-face interviews were not possible due to Covid restrictions. The interviews were audio recorded with the participant’s consent. The interviews were transcribed and then checked for accuracy and anonymised. The interviews lasted on average 60 min and were conducted between January to April 2021.

### Ethics

The study was approved by The University of Liverpool, Institute of Population Health Ethics committee (ID: 7636).

### Recruitment

We recruited for all arms of the study via our social media channels, personal Twitter accounts, and the Twitter account and general communication channels of the ARC NWC. We also sent direct emails to other NIHR organisations such as the NIHR Research Design Service and ARC national public involvement communities to ask them to distribute the survey link to their public contributors and PPIE professionals. The NIHR Centre for Dissemination and Engagement tweeted about the study. We also targeted charities and organisations involved in health and social care research asking them to distribute our study information to their public contributors and PPIE professionals. We had 244 respondents to the public contributor survey and 65 for the public involvement professionals (PIPs) survey and conducted 22 qualitative interviews (see Table 1). The public contributor survey included a final question on whether people would be interested in participating in qualitative interviews, and they could leave their email address for future contact.

**Table 1:** Overview of study participants

Demographics	Interviews—public contributors (%)	Survey—public contributors (%)	Survey PPIE professionals (%)
Number	22	244	65
Gender			
Male	13 (59.1)	102 (43.2)	56 (88.9)
Female	9 (40.9)	128 (54.2)	6 (9.5)
Mean age years (range)			
Age	62 (38–91)	63.2 (26–89)	44.5 (25–77)
Ethnicity			
Ethnic minority	7 (31.8)	28 (11.47)	3 (4.61)
White British	15 (68.2)	203 (83.2)	62 (95.4)
Numbers of years involved with PPIE			
0–1 years	2 (9.1)	16 (6.7)	11 (16.9)
1–5 years	5 (22.7)	102 (25.7)	41 (63.0)
> 5 years	15 (68.2)	121 (50.6)	13 (20)

### Data analysis

We drew on the growing literature on using online sources for both qualitative and quantitative research (Lupton [17]). Anonymised quantitative survey data were imported into SPSS version 27 (IBM, Somers, NY, USA) for descriptive inferential data analysis. Interview transcripts and the survey free text responses were coded by the team using NVivo 12 software to determine themes and key issues. Our analysis was interpretive, and we approached our data as both a resource and a topic [11]. The validity of the range of interpretations and suggested relationships between core themes was explored and tested against the data using the constant comparative method [28] and searching and accounting for deviant cases [29]. For the surveys, we used simple descriptive statistics to analyse the responses.

Our approach to integrating methods from qualitative and quantitative research were partly sequential, the data from the surveys helped us develop the topic guide for the qualitative interviews, but also at a data analysis level. To analyse our data, we adopted the ‘following the thread’ approach [18]. This approach to integration begins by analysing each data set, using the analytic strategies relevant for each type (see above), to identify key themes and questions, ‘to create a constellation of findings which can be used to generate a multi-faceted picture of the phenomenon. The value of this integrative analytic approach lies in allowing an inductive lead to the analysis, preserving the value of the open, exploratory, qualitative inquiry but incorporating the focus and specificity of the quantitative data.’ [, 54]. In this way, we developed themes within each individual data set and followed the thread across the other data sets. Hence, the data are presented in the results under thematic headings with figures and quotes from all three data sets (the two surveys and the qualitative interviews).^18^

### Reporting

We refer to those who took part in the survey as ‘*respondents*’, and the public contributors who took part in the qualitative interview as ‘*participants*’. After each quote we have included the participant number for the qualitative interviews and the unique identifier for the survey (see Table 2).

**Table 2:** Participant and respondent identifiers

Survey respondents		Unique identifier (generated by Jisc survey software)	Interview participants (1–22, for the 22 interviews)
Public contributors	PC	PC-70946561	PC-1
PPIE professional	PIP	PiP-74020148	N/A

For the percentages given for the survey responses, this is the percentage of those who answered the question not of the whole samples size. When the response rate for a question is very low, we will present the number of people who gave that answer.

## Results

### Background of the respondents

Most public contributor survey respondents (79%) worked with an NIHR funded organisation or research project, with 48% working with an NHS or social care organisation. This was also the case for the PIPs, with over half working for NIHR organisations (57%), on government-funded research (21.5%), and (41.5%) working with an NHS, social care organisation or public body. Respondents also worked with third sector groups and charities such as Cancer Research and the Alzheimer’s Society, with many public contributors working with more than one organisation. Most of the public contributors had been involved in PPIE for over 5 years, with only 7% being involved for a year or less. PIPs’ experience was evenly spread from 1 to 5 years, with just over half (52.4%) working part-time in their PPIE role. A quarter (25.4%) of the public contributors were from the least Index of Multiple Deprivation (IMD) Quintiles 1 and 2.^1^

Our themes can be summarised under the following headings, see Table 3 for an overview of the overarching themes identified through the analysis from both PC and PPI narratives and subthemes that are discussed in details below.

**Table 3:** Overview of themes in all the data

Theme	Sub-themes
Resources and financial compensation	Availability of resources Challenges to working from home Improvements to home working
Knowledge and information	Levels of digital literacy Confidence in using remote working tools
General support and training	Training provision Training preferences
The dynamics of working remotely	Satisfaction with video conferencing Discussing sensitive issues online Building and maintaining relationships Importance of chairing/moderating Clear code of conduct or/and house rules for online meetings More than one moderator is better for online meetings
Equality and diversity	Representation Improved accessibility for some Decreased accessibility for others
The future of PPIE meeting	

### Resources and financial compensation

#### Availability of resources

For participating in remote working PPIE, it is necessary to have the equipment needed to take part. In the survey, we asked people what kind of remote working equipment they had at home or had access to (see Table 4).

**Table 4:** What equipment do you have access to

Do you have access to	Public contributors	Public involvement professionals (PIPs)
Landline	91%	55%
Smart phone	88%	97%
Computer–desktop or laptop	96%	100%
Is this shared with anyone?	Yes shared—21%	Not shared—100%
Tablet	76%	66%
Internet at home	99%	100%
Internet at work	18%	44%

Most public contributors (77%) had a mobile phone contract, with 63% of those with contracts having unlimited calls and texts, and 30% having unlimited data. Of those who had limited calls, texts or data, most reported that this did not restrict their PPIE activities, as most used Wi-Fi and computers for their PPIE work.

Public contributors used a range of different electronic devices when doing remote PPIE. Some of the devices used had their limitations; for example, smart phones were not very practical in an online meeting, making it difficult to use all the functions and to see the meeting participants. Some respondents said that a lack of access to the internet was sometimes a problem, as they did not have enough credit on their phones to participate as much as they would like, with one respondent noting, ‘*I feel embarrassed to say I don’t have credit for example*.’ (PC 69251573).

#### Provision of resources by organisations

Most PIPs (77%) said that they had been provided with a computer to use at home since the start of the pandemic, and 10% had been given a financial contribution to telephone and internet charges. However, 63% had purchased equipment to help with remote working themselves, such as enhanced internet, an office chair, or another screen. A quarter of public contributors had bought new equipment to enable remote working for their PPIE activities, and had paid for it themselves, with only 4% reporting that their organisation had provided them with any equipment. Twenty-seven PIPs (34%) reported helping public contributors with phone and data costs, and five said they had provided equipment such as a computer or phone.

#### Challenges to working at home

Most public contributors (75%) reported that there were no aspects of their living conditions that limited their ability to do PPIE at home. Whereas 46% of PIPs reported that it was not ideal but they could manage, this view was expressed by just 17% of public contributors.

In both groups of respondents, the most common problems with working remotely were: poor internet connection, especially when others in the household were using the internet; noise; caring responsibilities; home schooling; lack of privacy when conducting meetings, particularly when discussing sensitive issues; lack of space, so having to use the kitchen table or living room—which created issues with noise and privacy; and having to pack away everything at the end of the day. Here a public contributor sums up the issues that they had experienced:

The only issue I have is when the whole thing freezes up, and my internet goes down. And then you have to re-join the meetings and things like that. And that is the frustrating thing. Because you can’t control the internet, if it decides to go off, it goes off. Or if my, my software decides it’s going to freeze and I can’t sort that out, I just have to go back and restart again. So that I’d say is the only really frustrating thing, which is totally outside your control. (PC-13)

#### Improvements to home working

When asked what you would need to work more effectively remotely, a large number of respondents in both groups agreed or strongly agreed with better internet connection (PCs = 44.4% and PIPs 74.2%). One respondent mentioned that they were homeless, and that clearly impacted on their ability to contribute to PPIE remotely, although they did report managing to be involved and finding the experience very beneficial for their mental health.

Public contributors emphasised the need to have access to resources, especially digital equipment. They also highlighted the need for additional equipment including cameras, headphones and a laptop to enable them to work remotely and fully contribute. One public contributor said it would be good to have ‘*a budget for hardware, software (office etc.) and for phone and internet top up, without feeling embarrassed or exposed*.’ (PC 70946561) Other items mentioned included appropriate chairs, screens and workspaces, so that people were able to use the technologies ergonomically, to reduce discomfort and not exacerbate existing health conditions.

It is important that remote working does not put a financial burden on the public contributor. With face-to-face meetings, public contributors are given expenses for travel and provided with refreshments, but remote working is not without costs to the public contributor, as one participant observed:

[the organisation] that I do stuff with have changed their payment policy, to include a remote working fee fixed remote working fee for meetings, if you attend a meeting, five pounds, or whatever it is, which I think really is really helped, because it doesn’t make you worry about, you know, if I have to use my phone, if for some reason the Wi Fi was down, I can use my phone on 4 g, and I’m not worrying about my day to day usage, stuff like that. (PC-8)

It is important to recognise that the shift to remote working was more difficult for some more than others, as observed by this participant:

The other issue, which for me is really important is the digital divide. Certain groups of people in that community are less likely to use this sort of media to get engaged. I think that the internet-based set of discussions will continue to disadvantage some groups of people. And that makes me very uncomfortable, because I’d like the PPI thing to be representative of the community that we’re serving. I don’t think we’ll achieve it while we’re based on the on the internet. (PC-5)

### Knowledge and information

#### Levels of digital literacy

We asked public contributors digital literacy questions, adapted from the Essential Digital Skills Framework produced by the Department of Education (DoE [7]). There are five key domains set out in this Framework: Communicating; Handling information and content; Transacting; Problem Solving; and Being safe and legal online.

In all domains, the majority of public contributor respondents reported a high level of digital literacy, with around 85% responding ‘I do this regularly’ to the majority of questions under each heading. They were confident in being able to complete tasks, such as back up and store data on a computer, search websites, purchase items online, and set appropriate privacy settings. The domain where they expressed least confidence was their ability to troubleshoot problems with a device or digital service using online help, with just 39% reporting that they did this regularly, (though a further 44% said that they had done this or could do this if asked).

Given the way our data were collected (see Limitations section), it is not surprising that 93% of our public contributor respondents reported using remote communication tools during lockdown, with 28% using them 1–2 times a week, 22% 3–4 times a week and 33% twice a month. Nearly half of the PIPs used remote communication tools 3–4 times a week.

#### Confidence in using remote working tools

Both public contributors and PIPs reported that their confidence in using remote communication tools had increased, with 44% of public contributors and 46% of PIPs saying, ‘quite a bit’. This was also reflected in the interviews with working remotely building public contributors’ confidence in trying new things.

Getting more involved means that you have more opportunities come your way. Like before I didn’t use social media as much either like WhatsApp and Facebook. More involvement has influenced me to use social media more as well. So now I use Twitter quite a lot, I use WhatsApp quite a lot. I think because this virtual platform has helped improve my skills on social media as well. (PC-14)

However, from the survey data, the public contributors’ confidence in using video conferencing tools such as Zoom and Microsoft Teams improved during Covid. Whereas, confidence in Skype and Facetime remained the same. This suggests that these had not been used very much as PPI remote working tools during the pandemic by our respondents.

The majority of public contributors (73%) reported that they liked remote working. The PIPs had more mixed views on this. Some reported that public contributors might not understand how to work remotely, with 32% of PIPs thinking some public contributors would have moderate to a lot of problems with remote working, and 41% thinking they would have some problems. Both public contributors and PIPs reported that their attitude to remote working had changed over lockdown and this change was largely in a positive direction, with people saying that it was easier than they had thought it would be:

But actually, a lot of the stuff I’ve been involved in, people have been absolutely brilliant. They’ve all learned new technologies and new ways of working, we’ve all done it together. And, you know, I think it’s been absolutely brilliant. (PC-8)

On the whole, 90% of the public contributor respondents in our survey felt they had coped well with working remotely. There was variation within this response, with 32% saying they could manage it but would prefer not to, and 14% saying it had not been easy. However, 62% said that they had been able to participate in projects and activities fully. The PIPs felt that their public contributors had adapted well to remote working, with 82% agreeing with this statement, and 81% saying that it could encourage greater public involvement.

### General support and training

#### Training provision

The rapid shift from face-to-face interactions to the use of remote working had often taken place without instituting any mechanisms of support for public contributors, though this is understandable given the reasons for this shift. Due to this, some public contributors felt that there was a risk of being excluded and that working remotely hindered their ability to participate in public involvement activities. For some, the use of digital communication (e.g., Zoom, Microsoft Teams) that they were not familiar with impacted on their confidence as public contributors:

I think during COVID 19 initially, the first few months I was excluded. Because I wasn’t confident on the platform. So, I wasn’t really aware, there was no training, no one had really informed us or explained anything. So, I found it quite difficult to build that confidence to ask and to use the zoom. And it was only through some of my other organizations, when they started to use the zoom, then I got involved. And then slowly, this was picked up, and we had some training. I found it quite difficult, the transformation from face to face to zoom. (PC-14)

During the early stages of remote working, public contributors had to act on their own initiative in learning how to use the new platforms of digital communication (e.g., Zoom) and develop their own understanding of how to contribute effectively in such settings.

Very different adjusting to doing things by Zoom, it was a sharp learning curve, trying to understand how these meetings are going to work. The necessary etiquette, the learning what the problems are with a technology bug, for example, the delays that go on and how difficult it is to have a conversation by remote when you’re used to meeting face to face. (PC-7)

Although formal training was limited, 73% of public contributors reported having received support from their organisation or project, with most finding this helpful, and 78% of PIPs reported providing support for their public contributors, such as Zoom training. For both public contributors and PIPs, the main way relevant new skills were acquired was through self-training online and then using skills learned this way repeatedly. Only around a quarter of both groups had received any training or induction from their organisations or workplaces. Help from family members was also mentioned as a way of solving problems:

My husband, whatever the problem I have he just sorted out. I only go to project team when obviously for other things, not for IT, because I know that my husband can sort them out. (PC-17)

#### Training preferences

Most public contributor respondents were keen to learn more, when asked ‘What would you need to work more effectively remotely? The respondents were provided with different options; Table 5 shows the % who agreed added to the % who strongly agreed with each option, for both groups of respondents. 55% (55.1%) of PCs agreed/strongly agreed with the training/knowledge option. They showed a preference for free online courses and materials provided by the health and social care research organisation, rather than face-to-face training and courses with a qualification. The biggest barrier for PIPs to provide training was financial: they reported that they had no budget for this.

**Table 5:** Responses to the survey question: ‘What would you need to work more effectively remotely?’

Option from survey	PCs (%)	PIPs (%)
Training/knowledge	55.1	73.7
Better internet	44.4	74.2
Provision of hardware	33.5	50
Adequate time for myself	30.3	50.8
Adequate space at home	26.1	47.5
None, as I do not want to work like this	8.6	3.8

Alongside training, participants noted that it is important to have access to individual support when needed. Some participants explained that having access to someone who could provide guidance or support, or direct them, made them feel confident in continuing their contribution and developing their capacity as a public contributor:

If I don’t understand it, I do ask that this help and support provided always and now as I become a public member co-lead [team name]. I’ve learned from the Team Manager, how I can use Teams. I asked her how I can do that. So, she provided help as well. I think if I asked for help, there’s always support and help available. (PC-18)

One theme emerging from the data was that public contributors felt they had been offered more support during the pandemic. They saw this as very valuable and wanted it to continue, as a participant commented:

So I think the support is there. It’s just hoping that they remember to keep the support once we go back to face to face. And everybody doesn’t get all busy and face to face meetings that they forget that we still need support, if we are using the virtual platform. (PC-8)

### The dynamics of working remotely

Video conferencing was the main way PPIE took place remotely. Most public contributors and all PIPs connected to remote video conferences via a computer/laptop, with 24% of public contributors using smart phones or tablets to connect. Generally, both groups of respondents reported video conferencing as easy to use and said that they had few technical problems.

#### Satisfaction with video conferencing

In terms of ease of use of video conferencing, public contributors found no difficulty in connecting to meetings, seeing everyone in meetings, being able to speak when they wanted, and being able to understand what was going on. Hearing people in meetings was harder, with the PIPs reported more difficulty in hearing everyone in the meeting with 59% of PIPs reporting some or lots of difficulty in hearing everyone and 51% of PCs reporting some or lots of difficulty. PIPs also reported slightly more difficulty in following and keeping track of what was going on (see Table 6). The respondents were provided with different options, what is recorded in this table is the % who reported some difficulty added to the % who reported lots of difficulty. Further, 55% of the PIPs reported that they had difficulty involving everyone in the meeting.

**Table 6:** Public contributor responses to the survey question: ‘*Overall, how user friendly have you found the following aspects of teleconference meetings?*’

Statement from survey	PCs (% reporting difficulty) (%)
Hearing everyone	58.6
Being able to speak when you want/need to	47.1
Understand what is going on	37.8
Follow and keep track of what was going on	48.2

Only 2% of public contributors did not like video conferencing, while 49% really liked it and 48% thought it was alright. There were no PIPs who did not like it, with 43% really liking it and 56% thinking it was alright. The majority of both PIPs and public contributors found it convenient, and PCs liked being able to do if from their own home. Most PIPs found video meetings tiring, whereas public contributors were split between those who did not (31%) and those who did (35%). Most respondents did not mind having their video on (PCs 78%).

The difficulties experienced in interacting online were explored further during the interviews with public contributors. With remote communication participants reported that the ‘human element’ of interaction was lacking and the ability to follow non-verbal communication that is often a key aspect of communicating was limited.

But it’s the ‘Have I said something stupid’, but in that in that sort of face to face, you very much get the personal feeling that no question is too stupid, that it’s safe to challenge. And the feeling of debate is more. it because it’s more sensitive, because you’re reading the body language, and you’re reading the, you know, the visual signs more. So yeah, so I think that’s, that was the main thing about the face to face. It was the it created that safe environment for somebody like me, who often will feel it’s the imposter syndrome is, you know, should I be here? Should I be doing this? Am I up to it? So they people help to put you at your ease. That’s what I think is what’s most noticeable. (PC-19)

Participants highlighted that it was difficult to ‘gauge reactions’, for instance whether others understood, acknowledged, and approved or disproved, when looking at small video on the screen.

If you sat around a table, you kind of pick up on other people’s cues, but it’s not so much, especially when there’s only kind of like a very tiny little screen. Sometimes you think, did they actually hear anything at all? Because you don’t get reply? (PC-1)

#### Building and maintaining relationships

There is some indication from participants that it might be more difficult to establish a relationship where one did not exist before and to maintain on-going engagement in a project with only online meetings. Many of the participants found it difficult to create relationships with others via remote communication; as one PIP said, ‘*Engagement, meetings, getting to know new communities doesn’t work well.*’ (PIP 71885453). They explained that face-to-face interactions promoted and eased building of relationships, as with face-to-face work they had the time outside of formal meetings to socially engage. For some, human interaction and building relationships was an important reason they were involved as a public contributor, and without it the motivation in being or continuing to be a public contributor is likely to reduce.

The other problem is that advantage of face to face, people can build relationship with one another. When you’re dealing remotely, it becomes artificial. That is what this remote working video conferencing telephone has done. It is breaking that relationship issue and if you don’t have that relationship issue, then that motivation of people getting involved in PPI, I fear will get reduced. That’s contentment, that satisfaction, that joy of taking part in PPI will get reduced. And most people who take part in PPI they’re not doing it for money, they’re doing it because they actually enjoy it and they feel it will be valuable for future generation. (PC-12)

There are other downsides with remote working: when asked what they missed by not having face-to-face meetings, public contributors said they missed seeing their colleagues at meetings and having informal chats. Reflections came both from PIPs, such as:

‘I realised the informal conversations are so important. Those small chats between a researcher and contributor when they are making coffee are just as important as the wider discussions.’ (PIP 71860940), and also this from a public contributor: ‘It has some uses but we must be careful not to over rely on this cheaper alternative to face to face. Humans interact face to face much more effectively for many tasks.’ (PC 70082446).

### Discussing sensitive issues online

**Fig. 1:**
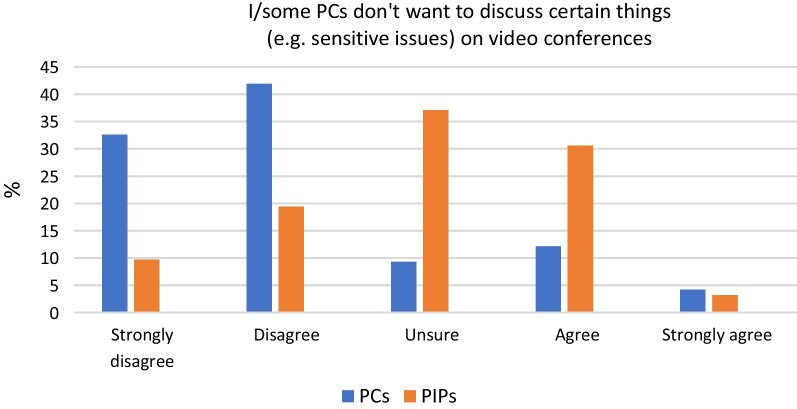
Discussion of sensitive issues

Public contributors said that they were happy discussing sensitive topics via video conference, and were not worried about online security or confidentiality, but 33% of PIPs thought that public contributors might not want to discuss sensitive topics online and 37% were unsure if they would (see Fig. 1).

Neither group of respondents were worried about recording the meetings, people listening in, or the organisation using their data.

#### Importance of chairing/moderating

Participants discussed the dynamics of remote/virtual meetings. They highlighted that remote meetings can often be difficult to run in comparison to face-to-face meetings, and require particular skills in a moderator or chair who can manage such an environment.

It has worked far less well when there has not been a good chair or moderator…. So do I put my physical hand up? Do I just unmute my microphone and shout? Or do I put my electronic hand up. And if I’m being good, and I mute my microphone, somebody else comes straight in and blurts out their point of view, and I’m thinking, hang on, they’re not being fair in they should have muted their microphone while I’m muting mine. So I don’t always feel it’s kind of that I know when to participate. And I sometimes feel irritable about other people who have just dived in when I when the chair has asked people to keep mute. (PC-21)

Public contributors explained that in online meetings there are different options for indicating when one wants to speak. As noted in the quote above, this includes raising a virtual hand, using the chat function, and also at times some will physically have their hand up to indicate their intention to contribute.

Participants explained that in a virtual setting it is important to create an environment that enables all members to contribute equally and is inclusive by managing ‘big characters’ who are likely to dominate discussions. They explained that when managing meetings, it is important to consider public contributors who may not have the confidence to contribute within a virtual group setting and may require support to enable them to fully contribute and feel included.

I think if you’ve got if you’ve got something to say then I think it’s even more important now then the person that’s holding the meeting, hosting the meeting, to be able to involve everybody and it’s really difficult. (PC-1)

#### Clear code of conduct or/and house rules for online meetings

Having a clear structure within meetings by clearly communicating the meeting ‘house rules’ or/and a ‘code of conduct’ (such as staying on mute while others are talking to avoid interruption and using the virtual hand function to indicate you wanted to speak next) helped manage the discussion and gave contributors confidence that their contribution will be acknowledged in an appropriate manner.

What works really well is if somebody says at the beginning, okay, ‘while I’m doing this presentation, everybody mute, if you have a comment, please put your electronic hand up. And I will look at it while the person is giving his presentation, I will look at the comments and the hands up and make sure you have a question’ that works really well. When we don’t have this, other times it’s muddles. (PC-21)

According to the participants, remote meeting facilitators need to ensure: a clear agenda; an introduction to members; communication at the start about when they are expected to contribute and when they need to remain on mute; and that there is time for all members to speak. It is also necessary to consider visibility on screen when sharing documents; the position of individuals on camera; and encouraging clear speech throughout the meeting. It is also useful to provide details on how to join or leave a meeting and describe how to use virtual backgrounds for privacy.

You have to go by the house rules of the room, if there was 10, or 20, people working, talking together, you got to control them. And naturally, we find it easier to control and speak one after another on various situation. So, we also find people who are invited to a meeting seems to know their roles within the organization, what to do, how to do it, and when to not interrupt anybody else, you know, respect to each other. (PC-11)

#### More than one moderator is better for online meetings

Participants found that to enable equal contribution, a meeting moderator needs support to manage the meeting; some highlighted that it is better to have several moderators rather than just one person trying to manage the meeting.

Really comes down to whoever’s organizing and running the call, especially if they have got two or three helpers, actually looking after it, e.g. muting everybody when you start. (PC-20)

This is especially important when the share screen function is used, which limits the ability to see all individuals on screen.

It worked well, when there’s been a good sort of facilitator/moderator, for example, what works really well is when a researcher is presenting their presentation, and there is another person who is looking out for people putting their physical hand up or their electronic hand up or making comments. (PC-21)

### Equality and diversity

As per our survey respondents and interview participants, diversity in PPIE generally is an issue. For example, only 18 (7.6%) of our PC respondents did not have English as a first language, and 83% self-identified as white British. We asked the PIPs to comment on the demographics of their public contributors and they reported that often public contributors are white, retired and middle class, although other groups were mentioned, and some PIPs worked with young people and people from groups who are traditionally underrepresented in health and social care research. We asked the PIPs if they thought the demographics of public contributors had changed during lockdown. One participant said,

And again, with the xxx they have a really, really strong young people’s group, the young people persons advisory group, and they are flying because the young people can fit it in from their universities or their homes in around their lives. And I think I think it will potentially open up the doors to make things more inclusive. (PC-19)

When asked if the demographics had changed, 47 respondents said no (77%), and 14 (23%) said yes. It was evenly split between some saying more ethnic minority people participating and some saying less; others said younger people were participating and others said less. So, it is not possible to infer any trends from this, as it appears to depend on the particular group and context.

In our study, most public contributors (83%) and PIPs (81%) reported that they had no reduced physical or mental abilities or health conditions that made using remote communication tools difficult (surveys data). Of those who reported difficulties, hearing loss, visual impairment and physical difficulties with managing computers were the most commonly mentioned. Roughly equal numbers of public contributors and PIPs reported difficulties (17% and 19%), and of these, 85% used assistive technologies to help with remote communication tools, such as voice recognition software and text to speech. While some public contributors reported not having anyone who they could ask for help with remote working (22.2%) no PIP respondents reported this.

#### Improved accessibility for some

We asked public contributors if their PPIE activities had increased during Covid: 58% reported that they had increased, 27% that they had stayed the same and 14% said they had decreased. PIPs also reported an increase in their PPIE activities (55%), with 16% saying they had decreased. 43% of public contributors said that their contact with their PPIE colleagues had remained the same during Covid (43%), but 35% reported it had increased and 20% that it had decreased (often due to studies running before Covid being mothballed). While 85% of PIPs thought that working remotely could make PPIE more inclusive and 44% of PIPs reported that they had had more contact with their public contributors, with 40% saying it remained the same and only two respondents reporting no contact.

I think this has become a blessing for me as it is so easy for me I don’t have to travel, I can easily log on, I’ve been taking part in so many opportunities, like it opened the door of getting involved. (PC-18)

There were some benefits to working remotely reported by both public contributors and PIPs. Remote working was viewed as having created more opportunities for PPIE, especially for those who found it hard to attend face-to-face meetings due to the physical aspects of having to travel. For some participants, remote worked addressed the physical barriers that had made them feel excluded.

[remote working] I think it’s opened up the area of PPI to a much wider scope of individuals and encourage them to take part where they wouldn’t have done before. I think that is beneficial to them because I suspect for the researchers, it’s given them access to a much wider audience of contributors than they would have had. (PC-20)

And the majority of people on the call were disabled people, including a visually impaired man, who has said that, since moving to online meetings, he thinks there’s been like an, he said, a 90% improvement for him. (PC-5)

Remote working was seen as convenient for various reasons, including being able to be involved from one’s own home, less travel and, consequently, less time and expense wasted. This gave an opportunity for greater involvement from certain sections of the community, who may not find it easy to travel to meetings to be engaged (those with disabilities, caring responsibilities, who did not have access to good transport).

‘It also allowed the discussions to happen with different contributors at times that suited them. As they have health problems, not having to travel was an advantage.’ (PIP 72095793).

However, participants reported that public contributors from communities where English is an additional language struggled the most, as issues such as language barriers played a greater role in limiting their access and contribution when PPIE was carried out online.

But then there’s certain projects that I’m a part of and sometimes when the moderator talks they’re not very clear talking very clearly. So you can’t really, you know, it’s difficult to hear what they’re saying. So if they can maybe speak a bit more clearly and also, when English is not your first language, there’s certain accents of English that you can get. And then there’s certain accents there are a bit hard for you to pick. So then you have to really listen a bit closely to what the other person saying. (PC-17)

#### Decreased accessibility for others

When carrying out PPIE remotely, participants reported different factors, such as age, learning ability or disabilities as potentially creating challenges when they are not acknowledged.

One of the big things for me with my hearing with deafness is that the problems created by other people are never deliberate. With the digital barrier there is a need to spend some time thinking about the people who read the rest of the screen. I think what happens with zoom is that when you start doing it, you’re not aware of how much energy it actually takes. If you are struggling to make sense of the words, and anyone with a hearing impairment is doing that. But at the end of our discussion now, I will be tired. I will take a break, have another cup of coffee. (PC-10)

Once this, once you go into screens, the face of the talker becomes a thumbnail. And I’m still using a lot of lip-reading assistance to understand people. (PC-10)

The physical aspects of remote working, such as the impact of sitting for long periods and looking at screens for people with muscular skeletal issues, were also raised:

Yeah. visual impairment, because I get double vision after a while. And if I’m concentrating, I also tend to get neck cramps, because I can’t sit for too long in one position, because of my back problem. (PC-12)

### The future of PPIE meetings

When asked how they would like meetings organised after the easing of pandemic-related social distancing measures, all participants and respondents reported that it is important to have flexibility to provide people with involvement opportunities that are suitable for them. It would be important to provide the opportunity to build relationships and support interaction through face-to-face working. Public contributors and PIPs did not want meetings to all be face-to-face. One public contributor said:

‘I fear that PPIE will expect us to only be present face to face after this, which would mean people like me would not be able to participate and isn’t fair. Many people with physical and neurological conditions benefit from this communication method.’ (PC 70946561).

Both groups favoured a combination of virtual and face-to-face meetings, and both agreed that remote working made it easier for some people to get involved in PPIE.

I would like a mixture. I would not like it always to be online. If we’re meeting between different locations online is good. But if it’s a few people in [city], a small group in [city] then I would like to travel and meet people I would feel that would be good for making friends and just better social contact with people. (PC-21)

As one PIP observed, ‘It [remote working] can be great for some people, but not all people. It’s not a one size fits all. Some people don’t like / or cannot effectively or easily access the required tools (e.g., no device, no reliable broadband, issues of digital exclusion). It can actively promote better inclusion - we have seen public contributors join meetings who would never join face to face sessions (they were not able to or did not like to travel), we have seen increased numbers (its quicker to join an hour video call than take half a day to join a meeting face to face). (PIP 74020148)

## Discussion

This study looked at how public contributors and PIPs had experienced carrying out PPIE remotely. While undertaking this study, two papers looking at remote PPIE were published [, 1, 16]. The findings from our study supports many of the insights from this work and adds to their contribution. On the whole, both groups, public contributors and PIPs, found working remotely manageable and in certain respects preferable to face-to-face meetings. Some of the main benefits of working remotely were that, for certain groups of public contributors, it increased accessibility of meetings and overcame various barriers to participating in PPIE activities. Those who found travelling difficult or who had caring responsibilities that made it hard to be away from home for long periods of time particularly welcomed working from home. However, there were limitations to this way of working; home environment, family, space, and internet connections all made home working challenging for some public contributors and PIPs. Both groups also missed the social and informal aspects of PPIE, which were hard to replicate online.

In their recent systematic review of patient involvement, Ocloo et al. [24] identified barriers and enablers to patient and public involvement. Although their review did not look specifically at remote access, many of the findings from our study fit into the key areas identified in their review, and we have organised our discussion to address these key areas. Ocloo et al.’s findings suggest that many of the challenges of remote access exist in PPIE more broadly.

### Resource and compensation

In remote access PPIE, it is crucial to ensure that the necessary resources, compensation and reimbursement mechanisms are in place. It is obvious, but important to note, that public contributors who lack the necessary equipment and ability to connect to the internet are completely excluded from remote PPIE activities. In our study, we found that only 4% of public contributors received any financial assistance towards equipment, and that a quarter of public contributors had purchased their own equipment to carry on contributing during the pandemic. It is likely that people with limited financial resources would have been unable to purchase their own equipment, making remote participation impossible. Even before the move to remote PPIE, people from low socio-economic status have been described as ‘hard to reach’ or ‘seldom heard from’ [10]. If the lack of digital resource provision remains unaddressed, then they will never be heard from.

Our study also suggests that PIPs were more likely than public contributors to be provided with equipment. This may indicate the undervaluing of public contributors, with some suggesting pre-pandemic that an asymmetry in compensation contributes to power imbalances (e.g., when the patient contributor does not receive any financial compensation, but the rest of the research team they are working with does) [27].

Some of the respondents and participants in this study suggested potential solutions to these issues of digital poverty (described as the lack of access to digital devices and ability to afford data plans and internet access) such as a fund for public contributors to apply to for equipment they need to fully participate, and a small additional renumeration for all public contributors towards the cost of data or Wi-Fi. Establishing these measures would hopefully avoid public contributors having to admit that they lack the resources to participate, which was described as a source of embarrassment.

Even when equipment is provided, our study also identified potential issues within participants’ home environments. Some, such as not having enough space to set up a workstation, are more difficult to address through financial payments; but others such as having caring responsibilities could also be remunerated, e.g., by covering the cost of a babysitter, during remote meetings. Some participants drew attention to physical difficulties, such as back troubles as a result of sitting for extended periods at online meetings; one suggestion to improve this would be to carry out a Display Screen Equipment (DSE) workstation assessment with public contributors who are regularly contributing remotely to reduce the risk of harm. Employers do this as part of health and safety legislation, however public contributors are not usually seen as employees and therefore not supported to the same extent and provided with safe home-working environments. Organisations could loan out suitable furniture for example from within their own stores, assuming it fell within fire and other health and safety/liability regulations to do so. This would mirror an expectation that attendees at face to face meetings would be offered the right facilities for their needs whether they were staff or invitees.

### Knowledge, support and training

Once equipment and internet access is in place it is also important to ensure that everyone has the knowledge and information necessary to participate remotely. Due to limitations in the study design (see the Limitations section), we did not capture the responses of those who have no or limited digital literacy. But it is important to ensure that support or training is provided so that people feel confident to participate in remote PPIE activities.

It is likely that PPIE organisers will need to provide training so that participants can develop the skills needed to participate fully. We found that public contributors looked to their organisations for training, online free courses, and one-to-one trouble-shooting support for particular problems. Formal or credit-bearing courses did not seem to be of interest to our respondents. One recommendation would be to carry out a skills and knowledge evaluation with current and new public contributors to identify areas for training and development. In our study, the domain where the respondents expressed least confidence was their ability to troubleshoot problems with a device or digital service using online help. This could be an area for skill development that could provide public contributors with the tools to solve technical issues when they arise. In terms of general support, public contributors welcomed what they saw as the additional support they had been offered during the pandemic, such as one-to-one phone calls and additional short catch-up meetings.

### Equality and diversity

It is hard to draw firm conclusions from our findings about the impact of remote working in PPIE on the diversity of public contributors who were involved during the Covid pandemic. In their study in doing PPIE with marginalised groups during the Covid pandemic, Adeyemi et al. [1] suggests that digital poverty is the biggest threat to inclusivity in remote working in PPIE. Other studies have also reported that digital exclusion is closely related to other forms of marginality [13]. The responses from the PIPs were mixed on this issue and given the limitations of the study (see below) it is likely that those who were digitally disenfranchised were unable to participate in remote working.

Just as when meeting in person for PPIE activities, remote access has a number of accessibility requirements to consider, which were identified in our study. The precise nature of accessibility requirements is, of course, dependent on the individuals that PPIE organisers aim to include, and remote PPIE activities need to be tailored for the specific participants who are attending. But some common considerations can be mentioned: the number of people in a meeting, the length of meetings, the screen display of documents, and the visibility of the meeting participants (especially for individuals who depend on lip reading) are all common issues. Adeyemi et al. [1] found that reaching out to organisations (e.g., UK charity the Royal National Institute of Blind people) for advice on how to tailor PPIE meetings to groups (in their case, people with a visual impairment) was particularly helpful.

Our study also found that some participants, especially those with English as an additional language, experienced an increase in linguistic barriers in remote meetings. Organisers of remote PPIE activities need to be aware of this potential barrier and consider additional support. Asking everyone to speak slowly and clearly at the start of an online PPIE activity has potential to benefit a wide range of participants, as we found that hearing everyone in online meetings presented difficulty for 51% of public contributors and 59% of PIPs. Appropriate accessibility software should be considered as part of the ‘equipment offer’.

### Representation

Ocloo et al. [24] note that issues of representativeness and lack of diverse perspectives are common concerns for PPIE. As we discussed earlier, it is likely that unless those organising PPIE put measures in place to tackle issues of digital poverty and digital literacy, then the move to remote PPIE will contribute increasing under-representation of some communities and groups. Yet as we found, while the move to online activities may decrease accessibility for those experiencing digital poverty, it has increased participation ability and made PPIE more accessible for other groups, such as those who cannot leave home due to illness.

Yetano and Royo’s [30] study on civic e-participation similarly found that online and offline participants had different socio-demographic profiles. As a result, they suggest that a combination of both forms of participation may lead to greater inclusion and representation. Similarly, our findings support the idea that a combination of online and face-to-face participation may lead to more inclusive PPIE.

### Power dynamics and organisational constraints

Karl et al. [15] suggest that videoconferencing may reduce the differences in status between meeting participants, since everyone appears in a box of the same size on some videoconferencing platforms. With this in mind, the move to remote PPIE might have been expected to help to address power asymmetries between ‘experts’ and the ‘public’, which some have found to be an issue in PPIE activities [25], and to have enabled public contributors to contribute more freely to discussions.

It was not clear from our data whether public contributors felt they were better able to express their opinions on videoconferencing, and a majority of public contributors in our study disagreed with the statement ‘I find it easier to say what I really want to when it’s not face-to-face’. Lampa et al. [16] found that public contributors shared less information in digital meetings. Our findings suggest that this is not due to concerns about sharing sensitive information, and public contributors did not express worries regarding privacy or confidentiality. Lampa et al. [16] suggests, rather, that public contributors find it difficult to claim their space online. The findings from our study might indicate this with some public contributors (47.1%) reporting difficulty in being able to speak when they want/need to in online video conferencing. In the interviews for our study, participants expressed some confusion regarding remote meeting etiquette and perceived unfairness regarding people speaking out of turn, which might also indicate a difficulty in claiming space.

Interview participants also highlighted the importance of the meeting’s chair for smooth running of online meetings and ensuring that everyone could participate and be fully involved, a finding that coheres with Lampa et al. [16], who note the increased importance of the chair/moderator in remote PPIE activities. This was also indicated in our survey data, with 55% of PIPs reporting that they had difficulty involving everyone in the meeting, and PIPs being more likely than public contributors to find video-conferencing tiring. One suggestion from our participants was to have multiple moderators running the meeting.

In the context of local politics, Fan and Fox [9] note that the move to remote civic participation has placed greater power in the hands of those organising activities, and correspondingly reduced the power of the public. Compared to in-person public meeting spaces, organisers of remote public meetings have more control over who can access the online space, who can contribute, and how they can do so. For example, online meetings involve the ability to mute participants, eject individual participants from meeting spaces, and turn the chat on or off.

These issues were not raised as a concern by participants in our study. Still, it is worth considering ways to mitigate this potential problem. Having a public contributor chair the meeting rather than a member of staff or a researcher and deciding on meeting etiquette and ground rules together as a group at the start, might be one way to reduce power imbalances that arise as a result of organisers’ and chairs’ increased responsibility in online PPIE activities. Lampa et al. [16] and Adeyemi et al. [1] both emphasise co-production of how online meetings will run as good practice. Given the increased importance of the chair’s role in online meetings, training could be considered to enable them to effectively fulfil the crucial role of ensuring everyone is fully involved in remote PPIE meetings.

There were also interesting differences in the responses of PIPs and public contributors, such as views on discussing sensitive issues and how tiring people found meetings. This emphasizes the need for PIPs to regularly check in with public contributors; our study found that what works better for PIPs in terms of online or face-to-face PPIE activities is not always what is best for public contributors; for instance, 83% of PIPs found video conferencing tiring compared to 46% of public contributors. However, it is worth noting that in some cases PIPs’ responses may reflect the wider PPIE group that they know and work with, rather than the technically confident public contributors who will have been responding to our online survey.

### Suggestions for the future of PPIE

Respondents and participants in our study favoured a combination of face-to-face and remote meetings; some were concerned about the potential move back to all meetings being face-to-face while others worried that face-to-face meetings would become a thing of the past. Adeyemi et al. [1] highlight that in-person contact remains a necessary strategy at the start of a PPIE journey to build trust and rapport, especially for those who are marginalised. In our study, most of the public contributors who responded to the survey had been involved in PPIE for over five years. Thus, many respondents may have already established relationships and built rapport before the move to online PPIE. Yetano and Royo [30] suggest that person-to-person contact dotted throughout a long-term collaboration is more effective at maintaining engagement; it is hard to tell from our findings what the long-term impact of online-only PPIE would have on maintaining participation. There were a number of good practice recommendations arising from our findings: the importance of a good moderator and/or chair to ensure everyone can participate fully; account for individual needs of public contributors when planning meetings; provide a small expenses payment alongside public contributor fees to cover phone/electricity or WiFi charges; and continue the individual support that was often offered to public contributors during the pandemic. These have been summarised in a freely available info graphic (https://arc-nwc.nihr.ac.uk/get-involved/opportunities/remoteworking/).

### Strengths and Limitations

The study took place during the early stages in the pandemic when the UK was undergoing lockdown measures Consequently, the study captures a snapshot of a particular time and how we worked.

A strength of the study is the number of respondents to the surveys and that we were able to follow up issues in the qualitative interviews. Further, both the surveys, with their free text response sections and the interviews gave public contributors and PIPs the opportunity to raise issues that had not been covered in the survey questions. The main limitation of our study was that due to lockdown lasting longer than originally anticipated when designing the study, we were unable to distribute the surveys as hard copies (as we could not access photocopying and university postal services), and we were only able to recruit those who had some form of digital access. Therefore, this study was not able to capture the views of those who were not able to be involved due to digital exclusion. Our findings have to be interpreted in the light of this, as all our participants and respondents were able to access digital resources. There were also some comments on Twitter that the survey was very long and took a long time to complete. We did pilot the survey with public contributors and tried to minimise the length, but it was a research study and we wanted to collect views on a range of areas of remote working. In the participant information sheet, we stressed that the survey was likely to take around 20 min.

## Conclusion

The Health Research Authority [14], when reflecting on PPIE during the pandemic, noted, ‘The pandemic has exposed and exacerbated the lack of resilience of the place of public involvement in UK research.’ Using remote working methods, in combination with face-to-face meetings, could be part of a strategy to improve PPIE across the health and social care research sector. By disrupting our traditional meeting format, remote working has focussed our attention on how meetings are run, their purpose, the advantages and costs of different meeting types, and how to work together more effectively. If done well, this ongoing critical consideration has the potential to make all types of PPIE meetings more productive and inclusive.

## Data Availability

The full data set, qualitative interviews and survey data is available on UK Data Service ReShare, Record 855,761 https://reshare.ukdataservice.ac.uk/.

## Footnotes

1 Multiple deprivation indices are datasets within the UK to classify the relative deprivation (essentially a measure of poverty) of small areas.
